# Transcriptomic Analysis of Neuropeptides and Peptide Hormones in the Barnacle *Balanus amphitrite*: Evidence of Roles in Larval Settlement

**DOI:** 10.1371/journal.pone.0046513

**Published:** 2012-10-02

**Authors:** Xing-Cheng Yan, Zhang-Fan Chen, Jin Sun, Kiyotaka Matsumura, Rudolf S. S. Wu, Pei-Yuan Qian

**Affiliations:** 1 Division of Life Science, The Hong Kong University of Science and Technology, Clear Water Bay, Kowloon, Hong Kong SAR, China; 2 Department of Biology, Hong Kong Baptist University, Kowloon Tong, Hong Kong SAR, China; 3 School of Biological Sciences, University of Hong Kong, Hong Kong SAR, China; University of Gothenburg, Sweden

## Abstract

The barnacle *Balanus amphitrite* is a globally distributed marine crustacean and has been used as a model species for intertidal ecology and biofouling studies. Its life cycle consists of seven planktonic larval stages followed by a sessile juvenile/adult stage. The transitional processes between larval stages and juveniles are crucial for barnacle development and recruitment. Although some studies have been conducted on the neuroanatomy and neuroactive substances of the barnacle, a comprehensive understanding of neuropeptides and peptide hormones remains lacking. To better characterize barnacle neuropeptidome and its potential roles in larval settlement, an *in silico* identification of putative transcripts encoding neuropeptides/peptide hormones was performed, based on transcriptome of the barnacle *B. amphitrite* that has been recently sequenced. Potential cleavage sites andstructure of mature peptides were predicted through homology search of known arthropod peptides. In total, 16 neuropeptide families/subfamilies were predicted from the barnacle transcriptome, and 14 of them were confirmed as genuine neuropeptides by Rapid Amplification of cDNA Ends. Analysis of peptide precursor structures and mature sequences showed that some neuropeptides of *B. amphitrite* are novel isoforms and shared similar characteristics with their homologs from insects. The expression profiling of predicted neuropeptide genes revealed that pigment dispersing hormone, SIFamide, calcitonin, and B-type allatostatin had the highest expression level in cypris stage, while tachykinin-related peptide was down regulated in both cyprids and juveniles. Furthermore, an inhibitor of proprotein convertase related to peptide maturation effectively delayed larval metamorphosis. Combination of real-time PCR results and bioassay indicated that certain neuropeptides may play an important role in cypris settlement. Overall, new insight into neuropeptides/peptide hormones characterized in this study shall provide a platform for unraveling peptidergic control of barnacle larval behavior and settlement process.

## Introduction

Neuropeptides constitute the largest class of intercellular messenger molecules and play key roles in many physiological processes, e.g. reproduction, homeostasis and locomotion [1]. In general, they are produced from endocrine cells or neurons as precursors (preprohormones) and become biologically active after post-translational modifications. Secreted neuropeptides can have autocrine, paracrine and hormonal effects, by binding to membrane receptors of organ systems [2]. The earliest traceable ancestral neuropeptides date back to primitive metazoans, i.e. cnidarians [3], [4]. In arthropods, neuropeptide studies have so far been limited to insects and decapods. For instance, eclosion hormone and ecdysis triggering hormone are the most well-known neuropeptides extensively studied in the moth *Manduca sexta* and the fruit fly *Drosophila melanogaster* [5]. Prothoracicotropic hormone has been characterized in various insects and proposed to initiate larval metamorphosis through stimulating prothoracic glands via G protein-coupled receptor/cAMP [6]. The crustacean hyperglycemic hormone family, originally isolated from X-organ-sinus gland (XO-SG) complex of decapods, was involved in regulating energy and ionic metabolism, or inhibiting molting and reproduction [7]. Besides insects and decapods, only very limited information is available on arthropod neuropeptides.

**Table 1:** Neuropeptide/peptide hormones predicted based on transcriptome mining of *Balanus amphitrite*.

Peptide family	Barnacle accessionNo.	Reference accessionNo.&	RACE confirmed	E-value	Score
**A-type allatostatin**	JQ864191	BAF64528.1	+	1E-22	1122
**B-type allatostatin**	JQ864192	NP_001036890.1	+	7E-22	249
**C-type allatostatin**	JQ864193	P85798.1	+	5E-23	94
**Bursicon α**	JQ864194	XP_003485714.1	+	3E-60	193
**Bursicon β**	JQ864195	ADI86243.1	+	5E-43	148
**Calcitonin-like diuretic hormone-isoform A**	JQ864196	EEZ99367.1	+	2E-14	71.6
**Calcitonin-like diuretic hormone-isoform B**	JQ864197	ACX47068.1	+	4E-21	90.5
**Corticotrophin-like diuretic hormone**	isotig15071#	UniRef100_P82373	−	0.005	36.6
**Eclosion hormone**	JQ864198	EGI68318.1	+	3E-11	64.7
**Insulin-related peptide**	JQ864199	NP_001233285.1	+	1E-08	57.4
**Ion transport peptide**	GBQDZ6L01AXT7W#	UniRef100_E0VME9	−	5E-19	89.4
	GBQDZ6L01BA9KU#	UniRef100_E2BEL2	−	0.003	37.4
**Neuropeptide F**	JQ864200	UniRef100_C9EAB8	+	0.005	36.6
**Orcokinin**	JQ864201	EFX70781.1	+	3E-27	110
**Pigment dispersing hormone**	JQ864202	JC4756	+	4E-14	70.1
**SIFamide**	JQ864203	NP_001161182.1	+	7E-06	47.8
**Sulfakinin**	JQ864204	NP_492344.2	+	1E-06	51.2
**Tachykinin-relate peptide**	GBQDZ6L01CRRE4#	BAD06363.1	−	0.0002	32
	JQ864205	BAC82426.1	+	3E-15	78.6

& Reference accession No. is the accession No. of the known neuropeptide that has the highest hit against barnacle transcript.

# NCBI accession numbers were not designated to sequences that were not confirmed by RACE.

Barnacles are common in intertidal communities worldwide, and often cause biofouling problems. The life-history of barnacles consists of six naupliar stages and one cypris stage when larvae become competent to attach to substratum and then metamorphose into sessile juveniles (collectively referred to as “settlement”). Cyprid is non-feeding stage and has evolved highly specialized features and behavior for settlement [8]. Besides the various exogenous inducers such as conspecific biogenic cues [9], [10], various biogenic amines and hormones such as serotonin [11], [12], dopamine, methyl farnesoate and 20-Hydroxyecdysone were also reported to regulate larval attachment and metamorphosis [13]–[16]. While extensive studies have been carried out on neurotransmitters/hormones in barnacle, only limited information is available on barnacle settlement.

Bioinformatics-based mining of neuropeptides from Expressed Sequence Tag (EST) library, transcriptome and genome has been recently conducted and numerous novel peptides have been uncovered in several species [17], [18]. The barnacle has been subjected to deep sequencing of EST recently [19]. In our study, we obtained the transcriptome of the barnacle *Balanus amphitrite* using 454 pyrosequencing technology, which contained more than 90,000 predicted open reading frames [20]. This rich source of transcriptomic information made large-scale *in silico* discovery of peptides feasible since we can overcome the difficulties of collecting and sectioning enough amounts of nervous tissues from *B. amphitrite* larvae for mass spectrometry analysis. In this study, we conducted *in silico* transcriptome mining of neuropeptides/peptide hormones in *B. amphitrite*, and quantified their expression levels at different developmental stages. We then examined the effect of proprotein convertase inhibitor on larval settlement to explore the possible function of the neuropeptides characterized. Our results provide a comprehensive catalog of neuropeptidome of *B. amphitrite*, and insights on the possible functional roles of some neuropeptides in barnacle larval settlement.

**Figure 1:**
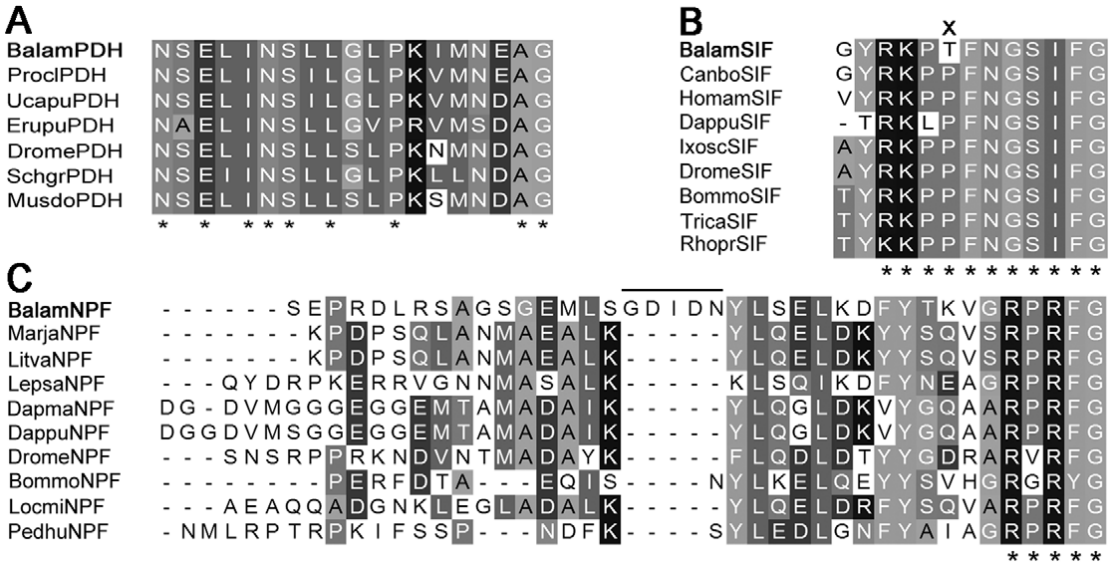
Sequence alignment of barnacle pigment dispersing hormone, SIFamide and neuropeptide F with arthropods. (A) Sequence alignment of mature pigment dispersing hormone. Decapods peptide sequences are from: *Uca pugilator* P08871, *Procambarus clarkii* Q9TWW7 and *Cancer productus* ABV68725; isopod peptide is from *Eurydice pulchra* ACX49752; insect peptides are from: *Drosophila melanogaster* AAL49303, *Schistocerca gregaria* ACY02888 and *Musca domestica* Q76JT9. (B) Alignment of barnacle SIFamide mature peptides. Sequences are from: decapods *Homarus americanus*, *Cancer borealis*; branchiopod *Daphnia pulex*; insects *Drosophila melanogaster*, *Tribolium castaneum*, *Bombyx mori*, *Rhodnius prolixus* and *Ixodes scapularis* [38]. (C) Alignment of barnacle NPF mature peptide. Insect NPF are from: *Drosophila melanogaster* AF117896, *Bombyx mori* NP_001124361, *Locusta migratoria* CO854418 and *Pediculus humanus* EEB15547. Decapods NPF are from shrimps *Marsupenaeus japonicus* CI998017 and *Litopenaeus vannamei* AEC12204; copepod NPF is from *Lepeophtheirus salmonis* FK933794; branchiopods NPF are from *Daphnia magna* EG565358 and *Daphnia pulex* EFX90018. Asterisks “*” represent typical motif residues of each peptide. “x” indicates unique amino acid residues of *B. amphitrite* compared to other arthropods. Line marks the unique five amino acids insertion of NPF in *B. amphitrite*.

## Materials and Methods

### 1. Sample Preparation

Adult barnacles were collected from the Pak Sha Wan public piers, Hong Kong (22°21′45″ N, 114°15′35″ E). No specific permits were required for the field collection. The field studies did not involve any endangered or protected species. Broods were isolated from adult barnacle in the laboratory and nauplii were hatched and cultured according to Thiyagarajan & Qian 2008 [21], and larvae were collected once they reached cypris stages after 4 days’ culture. For expression analysis of peptide precursor genes, nauplii II, nauplii VI, cyprids, newly metamorphosed juveniles, and adults were collected. Total RNA extraction and cDNA synthesis were conducted according to Chen et al. 2011 [20]. Briefly, total RNA of barnacles of different developmental stages was extracted with TRIzol® reagent (Invitrogen, Carlsbad, CA, USA). Trace DNA contaminants were removed by TURBO DNA-free™ Kit (Ambion Inc, Austin, TX, USA). cDNA was synthesized using M-MLV reverse transcriptase (USB, Cleveland, OH, USA) with oligo dT primer for Rapid Amplification of cDNA Ends (RACE) reactions and real-time PCR assays.

**Figure 2:**
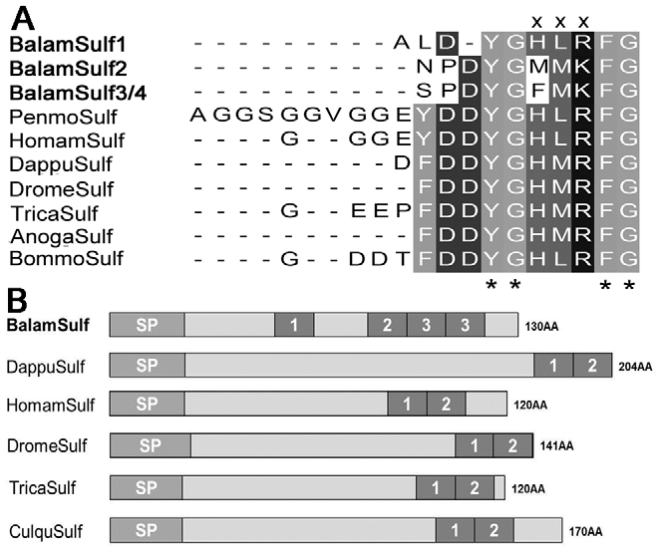
Comparison of barnacle sulfakinin peptide with other arthropods. (A) Sequence alignment of mature sulf. (B) Precursor structure of barnacle sulf compared with other crustaceans and insects. Insects Sulf are from: *Drosophila melanogaster* AAF52173, *Tribolium castaneum* EFA04708, *Anopheles gambiae* AAR03495, *Bombyx mori* NP_001124354 and *Culex quinquefasciatus* XP_001846221; branchiopod is from *Daphnia pulex* EFX80896; decapods Sulf are from *Homarus americanus* and *Penaeus monodon* [38]. Asterisks “*” represent typical motif residues of each peptide. “x” indicates unique amino acid residues of *B. amphitrite* compared to other arthropods.

### 2. Database Mining of Neuropeptide Precursors

Several methods from recent publications [17], [18], [22] were combined and modified to search for neuropeptide/peptide hormone encoding genes in barnacle transcriptome, which contains 23,451 contiguous sequences including 182 contigs, 23,269 isotigs and 77,785 singletons [20]. Protein sequences of the known neuropeptides and peptide hormones in arthropod were obtained from UniProt Knowledgebase (http://www.uniprot.org/) using “neuropeptide”, “hormone” and “peptide” as search keywords without "receptor", "signal anchor", or "transmembrane". NCBI non-redundant protein sequences (http://www.ncbi.nlm.nih.gov/) were also used for known arthropod peptides extraction, since different databases tend to use different key-word searching criteria which may lead to different results. After removing the unrelated sequences, such as enzymes and transcription factors, the remaining sequences were transformed into FASTA format to generate a local arthropod neuropeptide database. The program “tBLASTn” was used to mine for putative cDNA sequences that encode for active peptides in the barnacle transcriptome via queries using arthropod neuropeptide sequences mentioned above. For each query, the top three blast hits with an E-value lower than 0.01 were screened out and chosen as candidates and manually checked for homology to known peptides.

**Figure 3:**
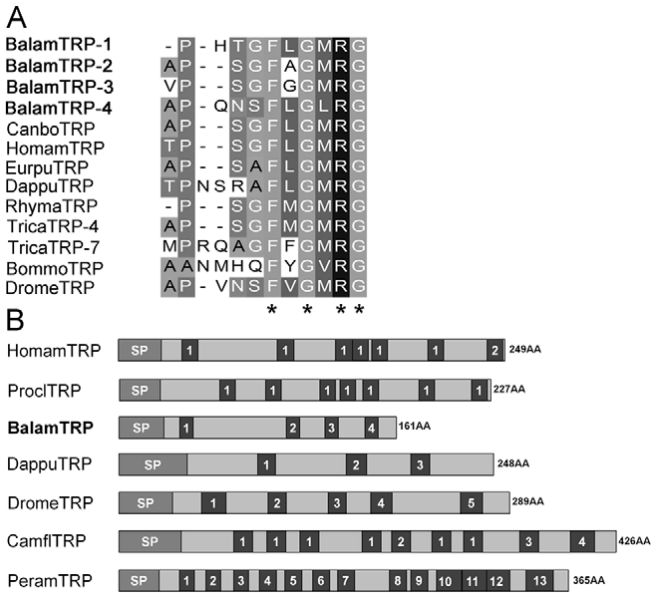
Comparison of barnacle tachykinin-related peptide with that of insects and crustaceans. (A) TRP sequence alignment. Decapods TRP are from: *Cancer borealis* [72] and *Homarus americanu*s ACB41786; isopod TRP is from *Eurydice pulchra* CO869025; branchiopod TRP is from *Daphnia pulex* [38]. Insects TRP are from: *Tribolium castaneum* EFA09176, *Drosophila melanogaster* AAF89172, *Bombyx mori* NP_001124364 and *Rhyparobia maderae* AAX11211. (B) Barnacle TRP precursor structure compared with other crustaceans and insects. Decapods TRP are from: *Homarus americanu*s ACB41786 and *Procambarus clarkia* BAC82426. Insects TRP are from: *Drosophila melanogaster* AAF89172, *Camponotus floridanus* EFN66667 and *Periplaneta americana* AAX11212. Branchiopod TRP is from *Daphnia pulex* [38]. Asterisks “*” represent typical motif residues of each peptide.

**Figure 4:**
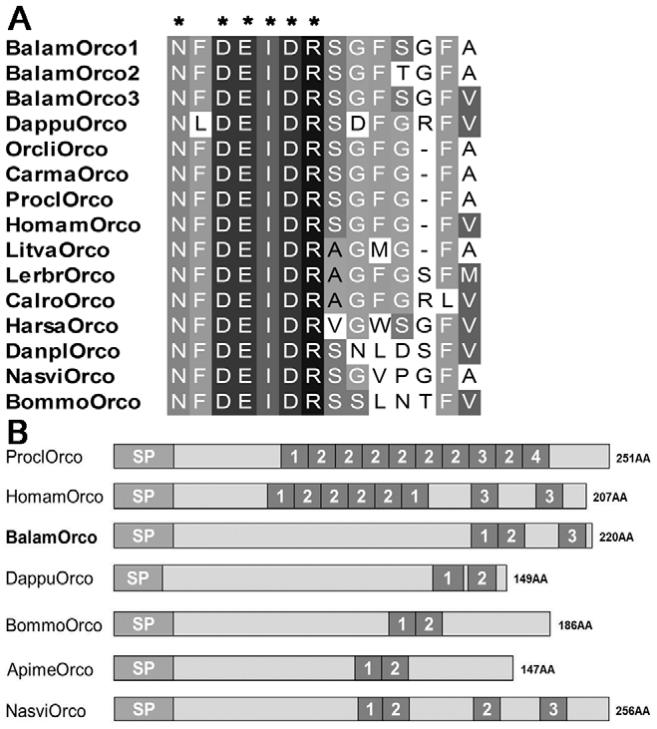
Comparison of barnacle orcokynin with other arthropods. (A) Sequence alignment mature orco. (B) Precursor structure of barnacle orco compared with other crustaceans and insects. Sequences of insects Orco are from: *Nasonia vitripennis* XP_003426062, *Bombyx mori* NP_001124366, *Danaus plexippus* EHJ77769, *Harpegnathos saltator* EFN80782 and *Apis mellifera* XP_001120650; copepods Orco are from: *Calius rogercresseyi* and *Lernaeocera branchialis* [22]; decapods Orco are from: *Litopenaeus vannamei* [1], *Marsupenaeus japonicas* [73], *Homarus americanus* ACD13197, *Procambarus clarkii* [73], *Carcinus maenas* [74] and *Orconectes limosus* [75]. Branchiopod Orco is from *Daphnia pulex* EFX70781. Asterisks “*” represent typical motif residues of each peptide.

### 3. Peptide Prediction

Neuropeptide candidate sequences generated by database mining were translated using ExPASy translate tool (http://web.expasy.org/translate/). Three typical neuropeptide precursor criteria, which are signal sequence, pro-hormone processing sites and length less than 300 amino acids, were applied to evaluate candidate sequences. Signal peptide identification was deduced by online program SignalP 3.0, using both the neural networks and Hidden Markov Model algorithms [23]. Pro-hormone cleavage sites were predicted based on work by Veenstra 2000 [24], and Neuropred online program (http://neuroproteomics.scs.illinois.edu/neuropred.html) and/or by homology to the known arthropod precursors. Sulfation state of Tyr residues was predicted using the online program Sulfinator [25] and/or by homology to known arthropod neuropeptides. In some cases, other post-translational modifications, e.g. cyclization of N-terminal Glu/Gln residues and C-terminal amidation were predicted mainly by homology to known peptide isoforms.

**Figure 5:**
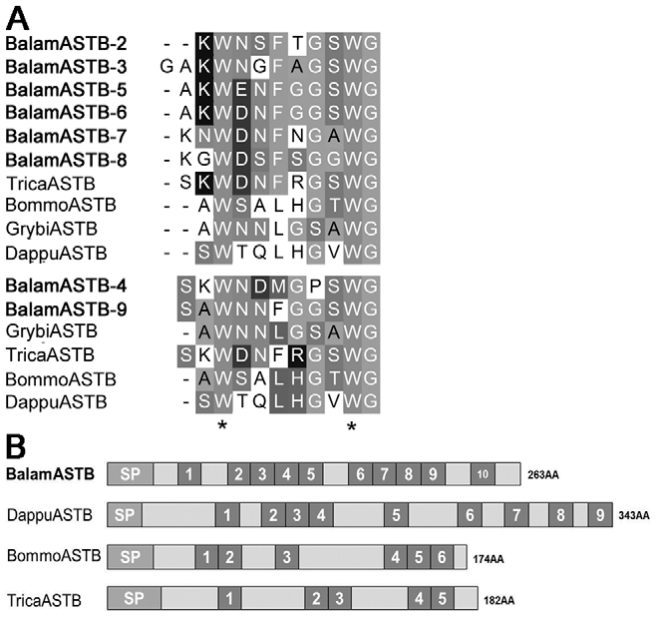
Comparison of barnacle B-type allatostatin with other arthropods. (A) Mature ASTB sequences alignment. (B) Precursor structure of barnacle ASTB compared with other crustaceans and insects. Insects ASTB are from: *Bombyx mori* P82003, *Tribolium castaneum* B8XQ58 and *Gryllus bimaculatus* Q5QRY7; branchiopod ASTB is from *Daphnia pulex* E9GSK4. Asterisks “*” represent conserved motif residues.

### 4. RACE Sequencing and Peptide Confirmation

Since neuropeptide precursor sequences generated from transcriptome are usually fragmented or incomplete, further confirmation on the predicted neuropeptide candidates by full length open reading frame (ORF) is required. Two sets of specific primers were designed from the partial cDNA sequences obtained from the transcriptome database. For RACE, a first run of amplification was performed using gene specific primer 1st and adaptor with oligo (dT)/oligo (dG) primer. Then gene specific primer 2nd (up or down to primer 1st) and adapter primers were used for second run PCR amplification. RACE products were cloned and sequenced. Complete amino acid sequences of candidate genes were submitted to NCBI BLAST again and checked manually for the precursor structure. If BLAST result of the new sequence didn’t match the corresponding neuropeptide genes, it was excluded.

**Figure 6:**
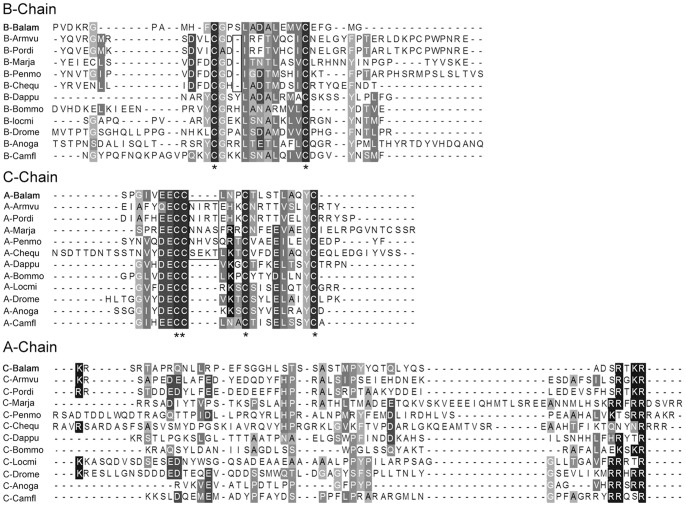
Peptide alignment of insulin-related peptide of *Balanus amphitrite* with arthropods. Isopods IRP are from: *Armadillidium vulgare* AB029615 and *Porcellio dilatatus* AB089811; decapods IRP are from: *Marsupenaeus japonicus* AB029615, *Penaeus monodon* GU208677.1 and *Cherax quadricarinatus* DQ851163; branchiopod IRP is from *Daphnia pulex* [38]; insects IRP are from: *Bombyx mori* NP_001233285, *Locusta migratoria* P15131, *Drosophila melanogaster* CG14173, *Anopheles gambiae* AAQ89693 and *Camponotus floridanus* EFN61735. Asterisks “*” mark the conserved Cysteine residues, black frame indicates specific amino acids insertion and deletion in IRP of isopod and decapod species, compared to insects, branchiopod and barnacle.

### 5. Sequence Analysis

Corresponding neuropeptide sequences from crustaceans and insects were searched and collected. All the crustacean neuropeptide genes and proteins discovered through *in silico* data mining, cloning or mass spectrometric approach were compiled. Predicted mature peptide sequences were used for alignment using ClustalW (version 2.0) with default parameters and manually checked.

**Figure 7:**
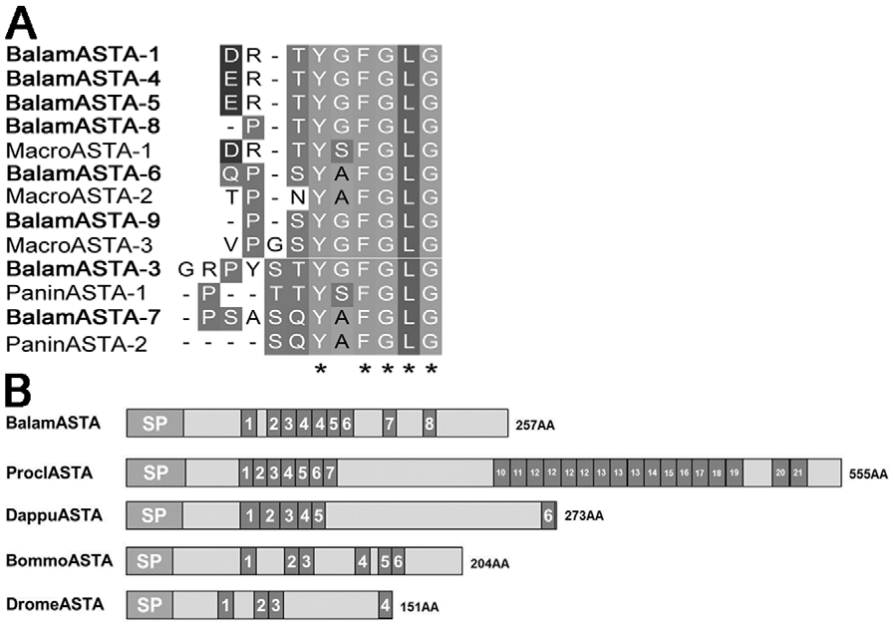
Sequence comparison of barnacle A-type allatostatin with homologs in arthropods. Decapod ASTAs are from *Macrobrachium rosenbergii* Q1AHE3, *Panulirus interruptus* A6BL33 and *Procambarus clarkii* Q3LI53; insect ASTAs are from *Bombyx mori* NP_001037036, *Drosophila melanogaster* AAF97792. Asterisk “*” represents typical motif residues of ASTA peptide.

### 6. Quantitative Real-time PCR

Gene specific primers were designed manually based on nucleotide sequences from the barnacle transcriptome database. Details of the primers are listed in Table S3 as additional information. The cytochrome b gene was used as the inner reference for normalizing the expression levels of target genes [26]. All real-time PCR assays for each peptide-encoding gene were performed on Stratagene Mx3005P QPCR System (Agilent, Santa Clara, CA, USA), using KAPA™ SYBR® FAST qPCR Kit Master Mix (2X) Universal (KAPA Biosystems, Woburn, MA, USA). For each neuropeptide gene, three replicates were conducted using each batch of larvae, and three batches of larvae were collected for real-time PCR analysis. Generated qRT-PCR Ct values were analyzed by 2-ΔΔCt method [27] and further tested by using one-way ANOVA, followed by Tukey test post-hoc analysis. Gene expression level in juvenile stage was standardized in order to better characterize genes that are up-regulated in cypris stage and down-regulated after metamorphosis, which might be involved in settlement regulation.

**Figure 8:**
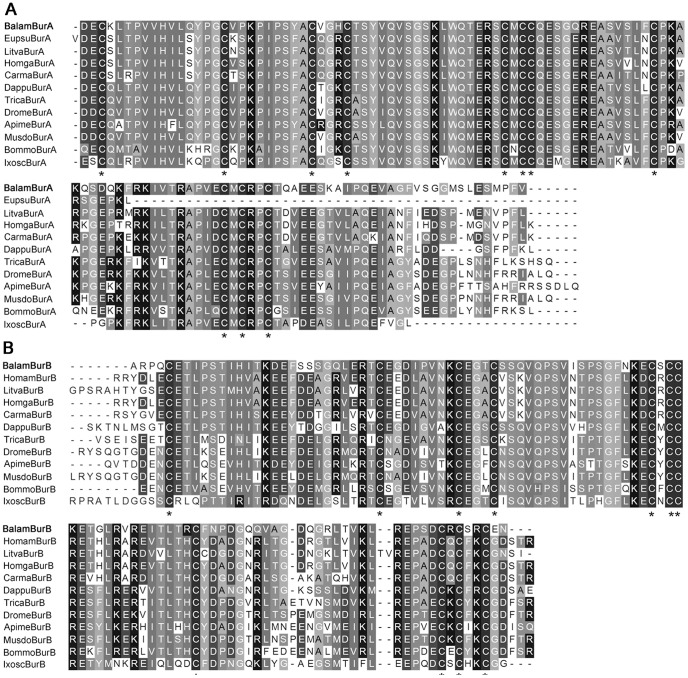
Sequence alignment of barnacle bursicon peptides. (A) Alignment of bursicon α subunit. Sequences are from: euphausiacean *Euphausia superba* [22]; decapods *Litopenaeus vannamei* [22], *Homarus gammarus* ADI86242.1 and *Carcinus maenas* EU139428; branchiopod *Daphnia pulex* [38]; insects *Tribolium castaneum* DQ138190, *Drosophila melanogaster* NM_142726, *Apis mellifera* NM_001098234, *Musca domestica* EF424614 and *Bombyx mori* NM_001098375; chelicerate *Ixodes scapularis* XM_002407468. (B) Alignment of bursicon β subunit. Sequences are from: decapods *Homarus americanus* [22], *Homarus gammarus* ADI86243.1, *Litopenaeus vannamei* [22] and Carcinus maenas EU139429; branchiopod *Daphnia pulex* [38]; insects *Tribolium castaneum* DQ156997, *Drosophila melanogaster* NM_135868, *Apis mellifera* NM_001040262, *Musca domestica* EF424613 and *Bombyx mori* NM_001043824; chelicerate *Ixodes scapularis* XM_002407469. Asterisk “*” mark the conserved cysteine that will form dimers.

### 7. Proprotein Convertase Inhibitor Assay

To test the hypothesis that neuropeptides/peptide hormones are involved in larval settlement, we incubated cyprids in solution of peptidyl chloromethylketone (Enzo life sciences, Farmingdale, NY, USA) and compared the percentage of metamorphose between the treatment and control. This compound is a highly specific and potent inhibitor against proprotein convertases responsible for maturation of bioactive peptides [28]. Specifically, stock solution (20 mM) was prepared by dissolving the compound in dimethyl sulfoxide (DMSO) and stored at −20°C. Experiments were conducted in triplicates and repeated three times with different batches of cyprids. Around 20 cyprids were added to each well of a 24-well plate (#3047, BD Falcon™, Franklin Lakes, NJ, USA) each containing 1 mL of test solution. The treatment group consisted of three concentrations (1, 10, and 100 µmolL^–1^), with two negative controls, i.e. autoclaved filtered seawater (AFSW) only and 0.5% DMSO in AFSW. The plates were incubated at 28°C in darkness for 48 hours, and the number of metamorphosed and swimming cyprids was counted under a dissecting microscope every 24 hours. Three replicates were conducted for each batch of larvae and treatment, and in total three batches of larvae were collected for bioassay. Percentage of larval metamorphosis was calculated and arcsine transformed prior to one-way ANOVA analysis followed by Tukey test post hoc analysis.

**Figure 9:**
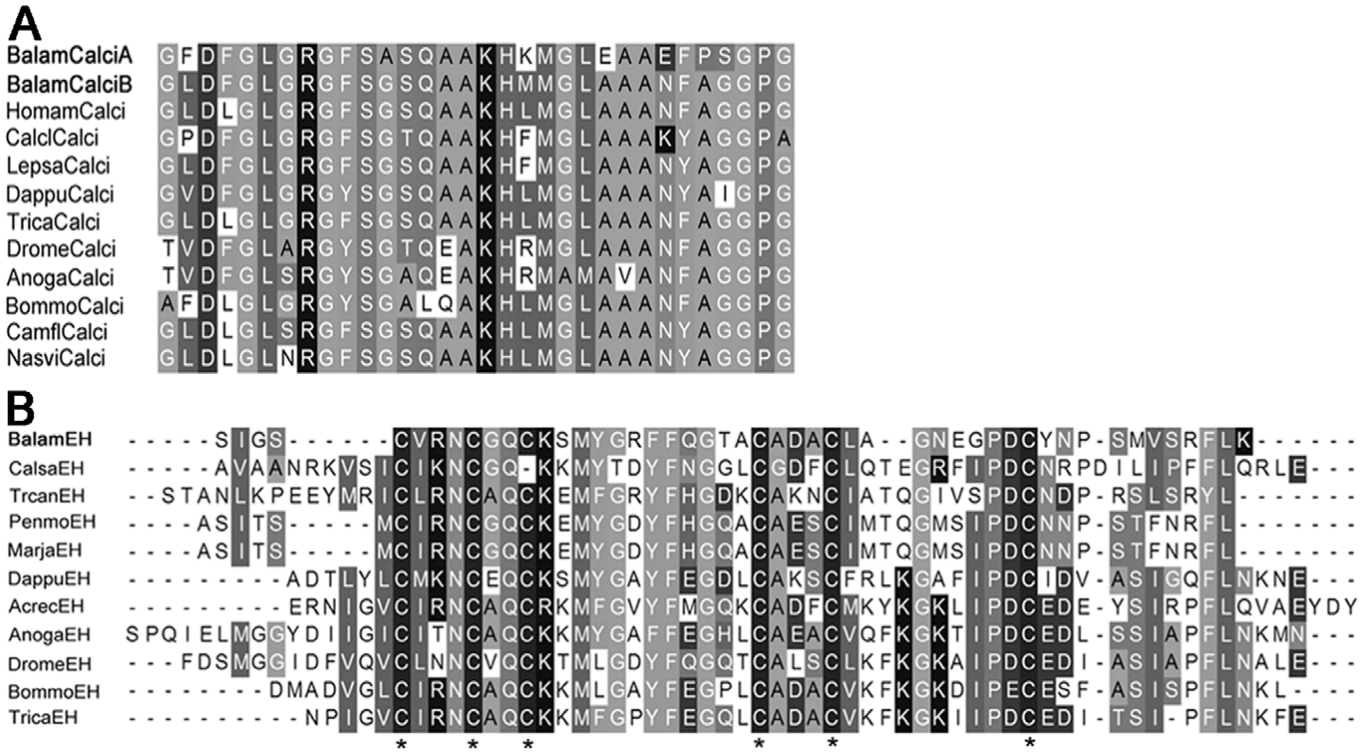
Sequence alignment of calcitonin and eclosion hormone of barnacle. (A) Sequence alignment of barnacle calcitonin isoform A and B. Sequences are from: decapods *Homarus americanus* ACX46386.1; copepods *Caligus clemensi* [22], *Lepeophtheirus salmonis* ADD38663; branchiopod *Daphnia pulex* EFX90445; insects *Tribolium castaneum* EEZ99367, *Drosophila melanogaster* NP_523514, *Anopheles gambiae* XP_321755, *Bombyx mori* NP_001124379, *Camponotus floridanus* EFN61187 and *Nasonia vitripennis* XP_001599948. (B) Alignment of barnacle eclosion hormone. Sequences are from: decapods *Callinectes sapidus* CV224237, *Penaeus monodon* [22], *Marsupenaeus japonicus* [22]; branchiopods *Daphnia pulex* [37], *Triops cancriformis* [22]; insects *Acromyrmex echinatior* EGI68318, *Anopheles gambiae* XP_001230805, *Drosophila melanogaster* NP_524386, *Bombyx mori* NM_001043842 and *Tribolium castaneum* XP_969164. Asterisks “*” mark the six conserved Cysteine residues in EH.

## Results and Discussion

### 1. Neuropeptides Predicted from *B. amphitrite*

In this study, a combination of *in silico* prediction of putative neuropeptide-encoding genes of *B. amphitrite* and molecular cloning verification were performed using known peptides in Arthropoda as queries. As a result, 16 neuropeptide families were predicted, and 14 of them were confirmed by peptide homology and gene cloning (Table 1), generating 64 predicted mature peptides (Table S1). Since many neuropeptides are relatively fragmented (10–30 amino acids) in the transcriptome dataset, the BLAST score tended to be low with a high E-value. Their full length open reading frames were cloned by RACE, which not only served as a secondary proof of the *in silico* predictions, but also provided large coverage of peptide isoforms as possible. The neuropeptide precursor sequences and predicted structures are listed in Table S2. In comparison with the previous studies in which only pigment dispersing hormone, crustacean cardioactive peptide and RFamide family had been characterized by immunohistochemistry [29], [30], [31], our results tremendously expand our knowledge of the molecular endocrinology of barnacle species.

**Figure 10:**
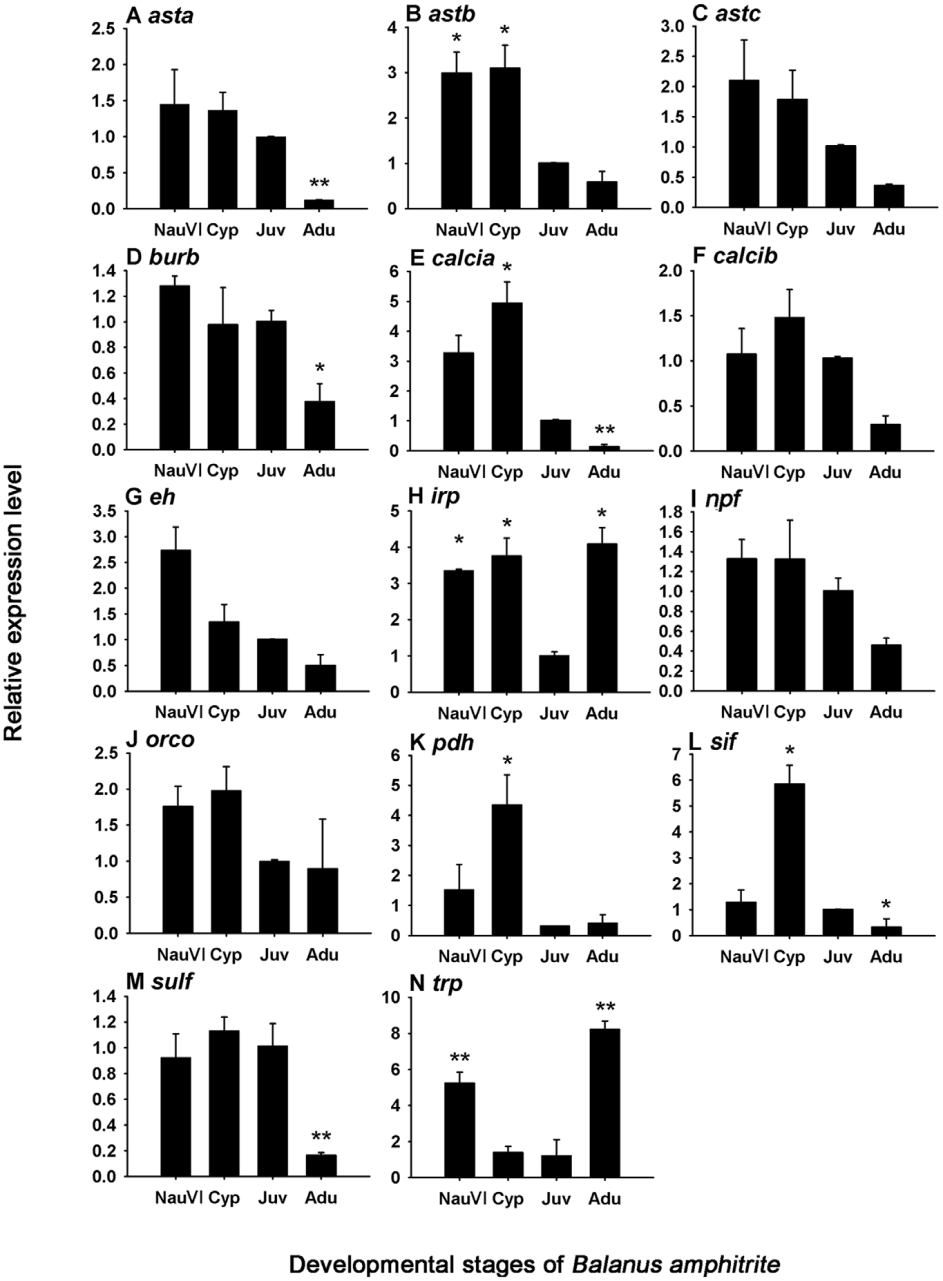
Real-time PCR results of predicted neuropeptide genes in four developmental stages. Stages include the late nauplius VI (NauVI), cyprid (Cyp), young juvenile (Juv) and the adult (Adu). Gene expression was measured for (A) A-type allatostatin (*asta*), (B) B-type allatostatin (*astb*), (C) C-type allatostatin (*astc*), (D) bursicon subunit β (*burb*), (E) calcitonin-like diuretic hormone isoform A (*calcia*), (F) calcitonin-like diuretic hormone isoform B (*calcib*), (G) eclosion hormone (*eh*), (H) insulin-related peptide (*irp*), (I) neuropeptide F (*npf*), (J) orcokinin (*orco*), (K) pigment dispersing hormone (*pdh*), (L) SIFamide (*sif*), (M) sulfakinin (*sulf*), (N) tachykinin-related peptide (*trp*). Values are showed as mean ± SD from three biological replicates. Asterisks indicate significant difference detected by one-way ANOVA comparing to gene expression level in juvenile stage (Tukey test, * p<0.05, **p<0.01).

The neuropeptides identified in this study included A-type allatostatin (ASTA), B-type allatostatin (ASTB), C-type allatostatin (ASTC), bursicon subunit α (BurA), bursicon subunit β (BurB), calcitonin-like diuretic hormone (Calci), eclosion hormone (EH), insulin-related peptide (IRP), neuropeptide F (NPF), orcokinin (Orco), pigment dispersing hormone (PDH), SIFamide, sulfakinin (Sulf) and tachykinin-related peptide (TRP) (Table 1). Among these predicted neuropeptides, pigment dispersing hormone was previously also detected in all of the three barnacle species tested by Webster 1998 [28]. In our *in silico* prediction, it was characterized with precise amino acid sequence as NSELINSLLGLPKIMNEAamide (Figure 1A, Table S1), which highly resembled β-PDH discovered in the crab *Uca pugilator* [22]. Two previously detected neuropeptides through immunohistochemistry, namely FMRF-like neuropeptide and crustacean cardioactive peptide (CCAP), were not found in our study. It might be because they were not covered by the transcriptome using 454 sequencing, or too fragmented to pass our screening criteria.

**Figure 11:**
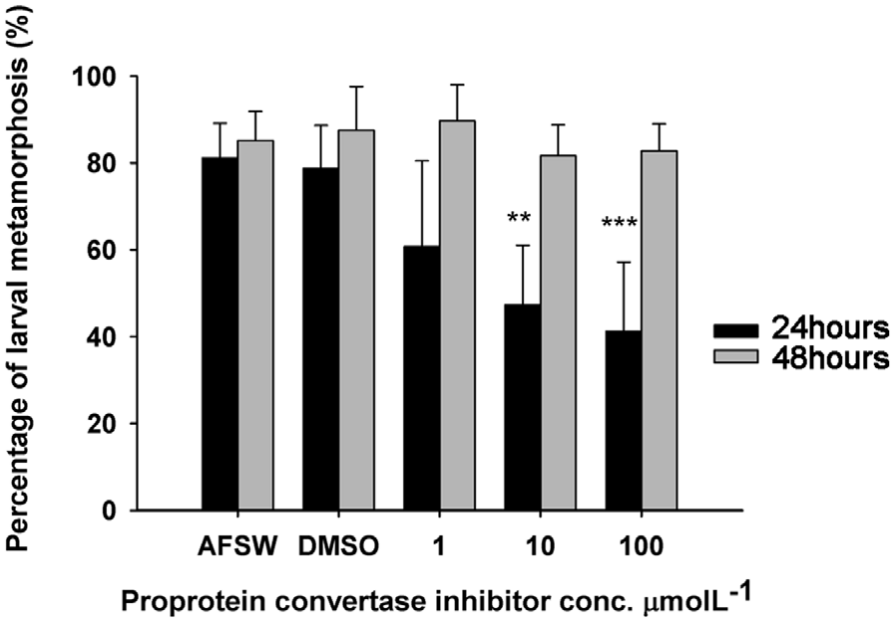
Proprotein convertase inhibitor bioassay result. Autoclaved filtered seawater (AFSW) and 0.1% DMSO served as positive control. Error bar represents mean ± SD for three biological replicates. Asterisks indicate significant difference detected by one-way ANOVA comparing the treatments to the positive control (Tukey test, **p<0.01 and ***p<0.001).

### 2. Neuropeptide Sequences Analysis

Although most of the neuropeptides identified in the present study are widely distributed among arthropod species, we found some isoform variants that appeared to be unique, including those that had previously been thought to be highly conserved in arthropods. For instance, SIFamide family consists of two major isoforms, namely Gly-SIFamide and Val-SIFamide, which are extremely conserved among arthropods and only differ at one N-terminal residues [32]. Gly-SIFamide peptide discovered in *B. amphitrite* in this study was highly similar to that of other insects and decapods, but with one amino acid Pro^6^ being changed into Thr^6^ (Figure 1B). Likewise, NPF of *B. amphitrite* possessed extra five-residues insertion between positions 19–23 from C-terminal, which was a novel isoform for NPF peptide family (Figure 1C). Furthermore, clear differences existed between sulfakinins from barnacle and their arthropod counterparts. The precursor of barnacle sulfakinin encoded for two more mature peptides than that of other arthropods (Figure 2B). In addition, compared with C-terminal typical signature YGHM/LRFamide with sulfated or nonsulfated Tyr in other species, sulfakinin-2/3/4 in *B. amphitrite* possessed Lys^8^ rather than the ubiquitous Arg^8^, and Met^6^/Phe^6^ instead of His^6^ in all known arthropod sulfakinin variants (Figure 2A). Further support was derived from barnacle ASTC with the mature sequence of SYWKQCSFNAVSCFamide (Table S1). The typical motif of ASTC is either X_6_CYFNPISCF with N-terminal blocked by pyro-Glu or SXWKQCAFNAVSCFamide [33], where X represents a variable residue. Thus ASTC of *B. amphitrite* highly resembled that of other decapods and insects, but with the broadly conserved Ala^7^ being substituted by Ser^7^.

During evolution, the non-synonymous mutations may either radical, or promote or impair the neuropeptide’s biological function [34]. Plenty of structural function studies on neuropeptides suggested even small variations of amino acid sequence can lead to substantial functional changes in the potency of the peptides, depending on the position of changes [35]. For instance, the allatostatin Pea-AST2 of the cockroach *Periplaneta americana* was reported to be more potent than Pea-AST1 over a 400-fold range, in terms of their ability of inhibiting juvenile hormone synthesis [36]. Thereby, unique neuropeptide structures found in barnacle may implicate that their functional efficiency has been altered. Whether, and in what way modified peptide sequences would alter their bioactivity and subsequently physiological processes in barnacles remains to be investigated.

### 3. Some Neuropeptides of *B. amphitrite* Indicate Closer Relationship with Insects Rather than Decapods

Insects and decapods are two major groups of arthropods widely studied in comparative endocrinology and neuropeptide physiology. Thorough comparison of neuropeptide structures and sequences revealed that some neuropeptides of barnacle were structurally similar to their insect homologs. The first instance was based on precursor structure of both the TRP and Orco genes of *B. amphitrite*. Decapods’ TRP precursor generally gave rise to several copies of a single TRP isoform or only one additional TRP isoform [37], while the TRP precursor of insects tended to encode multiple diverse isoforms [38]. The TRP precursor of *B. amphitrite* contained four copies of different TRP mature peptides (Figure 3, Table S2), which is structurally more similar to insects. In the case of Orco, mature Orco of both insects and *B. amphitrite* encoded for 14 amino acids, while the Orco of decapods was strictly 13 amino acids long (Figure 4A). Besides, comparison of their precursor structures also indicated that barnacle Orco resembled that of insects, with less mature peptides than crustaceans (Figure 4B).

The barnacle ASTB revealed in this study (Figure 5) lends further support to our postulation above. Among the 10 peptide isoforms encoded by ASTB precursor of *B. amphitrite*, 9 of them showed high similarity with the ASTB of insects. In addition, sequence alignment of IRP also indicated a similar result (Figure 6). In general, the IRP precursor contains contiguous B-C-A peptides. After maturation, A- and B-chain peptides are linked together by two interchain disulfide bonds and one intrachain disulfide bond. C-chain peptide assists the formation of linkage and will be clipped off at the cleavage sites afterwards [39]. IRP of *B. amphitrite* structurally resembled that of insects, with one more amino acid between the two Cys on B-chain and four amino acids gap between the second and third Cys of the A-chain (Figure 6). One contradiction came from the ASTA of *B. amphitrite*, which resemble that from decapods. Among predicted ASTA peptides of *B. amphitrite*, ASTA-1/4/5/6/8/9 were similar to ASTA peptides from the giant fresh water prawn *Macrobrachium rosenbergii*, while the rests shared similarities with the lobster *Panulirus interruptus* (Figure 7). Sequence comparison of other neuropeptides including BurA/B, Calci and EH were shown in Figure 8 and 9, revealing their conserved structures among arthropod species.

Based on molecular studies in the past decades, it is now widely accepted that hexapods are associated with crustaceans, forming a group called Pancrustacea within arthropods. However, the inner relationships among Pancrustacean constituent lineages are far from being resolved [40]. A recent phylogenetic analysis of protein-coding nuclear genes demonstrates that Hexapoda is most closely related to the crustacean Branchiopoda, Cephalocarida and Remipedia, while Malacostraca including decapods are grouped with Cirripedia (barnacles) making the traditional crustacean class Maxillopoda with copepod [41]. However in *B. amphitrite*, neuropeptides such as TRP, Orco, IRP and ASTB, were much similar to their homologs from insects rather than decapods, as supported by structure comparison and sequence alignment. High sequence similarity among different species may represent independent evolution under shared evolutionary constraints, which maintains structural and functional conservation of protein products [38]. The closer relationship of these neuropeptides between *B. amphitrite* and insects inferred much about their similar function in these two groups. Since molecular neuroendocrine information of arthropods other than decapods and insects is limited, data on neuropeptides from other taxa would be helpful.

### 4. Neuropeptides that are Specifically Up/down Regulated in Cyprid Stage

To further explore the potential involvement of neuropeptide genes in larval settlement of *B. amphitrite*, late nauplius VI, cyprid, early juvenile and adult were chosen to assess developmental variation of neuropeptide genes. Fourteen predicted neuropeptide genes were subjected to quantitative real-time PCR (Figure 10). Since the expression level of bursicon α subunit has already been measured by Chen et al. [20], this gene was omitted in the current developmental profiling. Using gene expression level in the juvenile stage as a standard, *calcia* (A isoform of calcitonin-like diuresis hormone), *sif* (SIFamide), *pdh* (pigment dispersing hormone) and *astb* (B-type allatostatin) were found to be specifically up-regulated in either late naupliar stage or cypris stage but down-regulated in early juvenile and adult stages (Tukey test, *p*<0.05). *trp* (Tachykinin-related peptide) was down-regulated in cypris and early juvenile stage compared to naupliar and adult stages (Tukey test, *p*<0.05); *irp* (Insulin-related peptide) was down regulated in early juvenile compared to other stages (*p*<0.05, One-way ANOVA); expression level of *npf* (neuropeptide F), *burb* (bursicon subunit β), *calcib* (B isoform of calcitonin-like diuresis hormone), *orco* (orcokinin), *sulf* (sulfakinin), *eh* (eclosion hormone), *asta* and *astc* (A- and C- type allatostatin) remained unchanged among naupliar VI, cypris, juvenile stages, but were down-regulated in adults. The down regulation of neuropeptide genes in adults could be due to the degenerated neural system since the central nervous system of adult barnacle is highly reduced in the sessile mode [29], [42]. There was, however, an exception, which is the high expression of tachykinin-related peptide (*trp*) in both naupliar VI and adult stages. Since the nervous system of cyprid larva is specifically cater for sensing settlement cues [43], we thus expect that peptide B-type allatostatin, A isoform of calcitonin-like diuresis hormone (Calci-A), pigment dispersing hormone and SIFamide are involved in cypris attachment and metamorphosis, or at least performs a specific function during these two stages, since they were higher expressed in larval stage but down-regulated after metamorphosis.

#### 4.1 B-type allatostatin

All three families of ASTs were found in the transcriptome of *B. amphitrite*, but only ASTB was up-regulated in both naupliar VI and cypris stages, being almost 3-fold higher than the juvenile stage (Figure 10B). Different developmental expression patterns of these three types of ASTs in *B. amphitrite* indicates that each type may be respectively involved in different physiological processes, such as juvenile hormone synthesis, stomatogastric or cardiac neuromuscular functions reported in insects [44], [45]. One major function of ASTs in the insects studied is inhibiting juvenile hormone (JH) synthesis [46]. JH is responsible for the maintenance of juvenile characteristics during development, and prevents metamorphosis during larval stage. Methyl farnesoate (MF), the unepoxidised form of JH III discovered in crustacean, was recently considered as functionally equivalent to insect JH [47]. In barnacle, high concentration of exogenous MF induced precocious metamorphosis without attachment [14], while physiologically-relevant concentration of natural isomer of MF inhibited larval settlement [16]. Furthermore, Yamamoto et al. [13] suggested that the balance of JH and 20-Hydroxyecdysone could regulate cypris metamorphosis, especially molting. Since ASTB was reported to have JH-inhibiting effects in other species, we presume that high expression level of ASTB in late cyprid stage would suppress JH synthesis and subsequently regulates metamorphosis.

#### 4.2 Calcitonin-like diuretic hormone

In our study, two isoforms of calcitonin-like diuretic hormone (CalciA and CalciB) located at different transcripts were identified and distinct expression patterns were detected. CalciA was highly expressed in both Nauplii VI and cyprid compared with juvenile stage, while CalciB’s expression didn’t significantly change during settlement (Figure 10E, F). Calci belongs to the diuretic hormone (DH) family and could promote fluid secretion of Malpighian tubule in insects [48], acting as mosquito natriuretic peptide that can stimulate Na+ rich urine [49], or modulated diuresis-related hindgut activity [50]. In crustaceans, Calci was characterized in the American lobster *Homarus americanus* and functioned as an intrinsic modulator of cardiac output [51]. Thus barnacle CalciA may be involved in maintaining ionic homeostasis of hemolymph during barnacle development.

#### 4.3 Pigment dispersing hormone

The expression level of PDH was 4-fold higher in cyprid than that in juvenile (Figure 10K). Crustacean PDH is homolog to pigment dispersing factor (PDF) discovered in insects. In insects, PDF served as a major output signal in the biological clock for fruit fly and cockcroach [52]−[54], which in turn regulated physiological processes and behavior related to daily rhythms. PDF was also located at visual interneurons in the synaptic neuropil (lamina) underlying the compound eye of the housefly *Musca domestica* [55]. In crustaceans, PDH has been reported to induce pigment movements in chromatophores and retinal pigment cells [56], or affected electrical response to photic stimulation of the compound eyes [57]. According to Webster 1998 [30], strong PDH immunoreactivity was found in perikarya on the surface of the neuropil of the ventral ganglion and supra-esophageal ganglia in adult barnacle species, and thus PDH was suggested to have neuromodulatory roles in somatic extensions in adult barnacles. At this moment no information is available for localization of the PDH in barnacle larvae. The nervous system of cyprid is more complicated than adult, and only cypris larva has a pair of morphologically well-differentiated compound eyes. Higher expression of PDH in the cypris stage and its general function in vision suggest that it may be related to the photoreception of compound eyes in cyprids during larval settlement.

#### 4.4 SIFamide

The expression level of SIFamide was nearly 6-fold higher in the cypris stage than in juvenile (Figure 10L). SIFamide peptide family is broadly distributed among arthropod and highly conserved. It has diverse functions and acts as a local autocrine/paracrine modulator. In *Drosophila*, SIFamide could modulate sexual behavior [58], while in crustaceans, it was related to dominance hierarchy of the prawn *M. rosenbergii* [59], or modulating pyloric neural circuit in the lobster *H. americanus* [60]. Immunohistochemistry work showed that SIFamide was densely accumulated in the olfactory lobe in the crayfish *Procambarus clarkii*, indicating its function in olfactory systems [59]. Another study confirmed the presence of SIFamide in the eyestalk neuropils of a crayfish and suggested its role in visual signal processing [61]. The choice of substratum for permanent attachment of competent cyprid relies on sensitive response to both physical and chemical characteristics of environment as well as conspecific biogenic cues [62]. Since SIFamide is related to processing high-order, multimodal input and transmitting tactile, olfactory and visual stimuli [32], a higher expression level in cypris stage is required for transmitting neural signals and detecting exogenic cues in the settlement processes.

#### 4.5 Tachykinin-related peptide

Expression of TRP in barnacle was down-regulated in the cypris and juvenile stages, compared to its relatively high expression in naupliar VI and adult stages (Figure 10N). The TRP family represents one of the largest neuropeptide families in the animal kingdom and is widely distributed across invertebrate, protochordate, and vertebrate species [63]. Previous researches suggested that TRP might function as both central neuromodulators and circulating hormones [64]. The TRPs display multiple functions in the nervous system and different kinds of muscle, and most importantly in gut tissue among insects [65]. TRP is related to feeding status in locust evident by a decrease in immunoreactivity after 48 hours of starvation [66]. In crustaceans, TRP was first discovered in the crab *Cancer borealis* and exactly the same sequence was then found in other seven crab species [37]. In general, TRP is related to food intake and digestion related functions. Since the cyprid larvae do not feed, the subsequent habitat selection and settlement behavior are dependent on energy reservation [67], i.e. lipids and vitellin-like protein [68]. During settlement, barnacle larvae undergo tremendous morphological changes and begin to feed from 2 to 5 days afterward [69]. TRP expression attained a relatively low level in the two non-feeding stages, cyprid and early juvenile, indicating its paracrine/hormonal control of feeding-related behavior of barnacle.

### 5. Prohormone Convertase Inhibitor Effectively Delayed Larval Settlement of *B. amphitrite*

Neuropeptides are derived from larger proprotein precursors which carry one or more mature peptides. Highly regulated posttranslational transformation is required for generating mature peptides with biological functions. After cleavage of N-terminal signal peptide, proprotein convertase (PC) cleaves at the mono- or diabasic cleavage sites of the remaining part of precursor, giving rise to peptide products that will undergo subsequent peptidase modification [70]. Two members of proprotein convertase family, PC2 and PC1/PC3, appear to play a preeminent role in neuroendocrine precursor maturation process in both mammalian and invertebrates [71]. In *Caenorhabditis elegans*, HPLC-MALDI-TOF analysis indicated a drastic reduction of types and abundance of neuropeptides in KPC-2/KPC-3 (PC homologs) mutant strains compared to wild type strains [70]. The *kpc-2*/*egl-3* mutant was still viable, but its responsiveness to mechanical stimuli and egg-laying behavior were impaired [70].

To further explore peptidergic control of larval settlement of *B. amphitrite*, we performed settlement This inhibitor is the most potent commercial compound that specifically inhibits peptide production and maturation [28]. Bioassay result showed that this inhibitor effectively delayed larval attachment and metamorphosis of *B. amphitrite*, on a dose dependent manner. After 24 hours, larval metamorphosis was significantly inhibited when the inhibitor concentration ≥10 µmolL^–1^ (Tukey test, **p<0.01, ***p<0.001), compared with cyprids incubated in AFSW or 0.5% DMSO as the control, while no significant effect was observed at 1 µmolL^–1^ (Tukey test, *p* = 0.081). The inhibition was unlikely to be caused by toxicity of PC inhibitor since the unsettled cyprids in treatment group were swimming normally. After incubation for 48 hours, most of the swimming cyprids in the treatment group settled and metamorphosed normally into early juveniles, and larval metamorphosis percentage among the controls and treatments was not different (Tukey test, *p*>0.05) (Figure 11). No mortality was observed for all the tested concentrations within the experimental duration. We may deduce that PC inhibitor restrained peptide maturation and thus cyprids delayed metamorphosis into juvenile. The real concentration of the compound in cyprids might be lower than the nominal concentration [11], and the peptide may be degraded by enzymes in hemolymph 48 hours after treatment. The complimentary peptide maturation pathways in addition of PC could be another reason why settlement was not completely blocked by inhibitor.

### Conclusion

In conclusion, we discovered fourteen neuropeptide and peptide hormone families/subfamilies through *in silico* transcriptome mining of *Balanus amphitrite*. The analysis of mature structure and sequence of the predicted neuropeptides provided a new evidence on evolution of barnacle neuropeptides. B-type allatostatin, calcitonin, pigment dispersing hormone and SIFamide were up-regulated in cypris stage and down-regulated after metamorphosis. Together with our bioassay result of proprotein convertase inhibitor, we demonstrated the involvement of neuropeptides in larval metamorphosis. Our neuropeptidome data also provide a platform for further elucidating the physiological functions of individual peptide. Specifically, synthetic peptide could be raised based on the predicted peptide structure, for exploring their spatial expression pattern through specific antibodies, or for *in vivo* test of their functions in barnacle through peptide treatment. Given that *B. amphitrite* is an important biofouling species worldwide, neuropeptide genes and their postulated functional role in larval settlement revealed in this study may shed light on the future development of novel antifouling compounds.

## Supporting Information

Table S1 Mature neuropeptides/peptide hormones predicted from *Balanus amphitrite*.
(PDF)

Table S2 Precursor sequences of neuropeptides and peptide hormones from *Balanus amphitrite*. Signal peptides are marked as green letter, precursor related peptides are marked as blue letter, and mature neuropeptides are marked as red letter. The putative mono-, di- or tribasic cleavage sites are underlined, and amino acid residues that predicted to be sulfated are shaded as pink.
(PDF)

Table S3 Primers used for real-time PCR amplification.
(PDF)
