# Supplementary material for: Transcriptomic Analysis of Neuropeptides and Peptide Hormones in the Barnacle Balanus amphitrite: Evidence of Roles in Larval Settlement
Source: PLoS One. 2012 Oct 2;7(10):e46513. doi: 10.1371/journal.pone.0046513 (PMC3462748; doi:10.1371/journal.pone.0046513)
Supplement: Table S1 — Mature neuropeptides/peptide hormones predicted from Balanus amphitrite. (PDF) [file pone.0046513.s001.pdf]

Table S1. Mature neuropeptides/peptide hormones predicted from *Balanus amphitrite*.

Peptide name	Predicted mature peptide
**ASTA-1**	DRTYGFGLa
**ASTA-2**	DQNRPYRAYGFGLa
**ASTA-3**	GRPYSTYGFGLa
**ASTA-4**	ERTYGFGLa
**ASTA-5**	ERTYGFGLa
**ASTA-6**	QPSYAFGLa
**ASTA-7**	PSASQYAFGLa
**ASTA-8**	PTYGFGLa
**ASTA-9**	PSYGFGLa
**ASTA-PRP1**	KQLGQDHQLATDLLDDNYDDTY(SOH)DLEPDGEDVE
**ASTA-PRP2**	NGHDDIPN
**ASTA-PRP3**	SWSWFEGPTRDVL
**ASTA-PRP4**	SAPAGVDEDLLDDPDTALLMTAARDLADGEVKSPAGRHRIGSDSGQTHTKPEDVSSQEKGVRFQL
**ASTB-1**	DWNALHGNWa
**ASTB-2**	KWNSFTGSWa
**ASTB-3**	GAKWNGFAGSWa
**ASTB-4**	SKWNDMGPSWa
**ASTB-5**	AKWENFGGSWa
**ASTB-6**	AKWDNFGGSWa
**ASTB-7**	KNWDNFNGAWa
**ASTB-8**	KGWDSFSGGWa
**ASTB-9**	SAWNNFGGSWa
**ASTB-10**	RKDWTQLNGMWa
**ASTB-PRP1**	DPSEVTGDPEDDRELMREVANGLVLSAYEPED
**ASTB-PRP2**	SKWDDIDDDSLE
**ASTB-PRP3**	SVSTPELQEAADRLEKFIVSHYEPSPEVEVSSGETQA
**ASTB-PRP4**	SSSKALLLA
**ASTC**	SYWKQCSFNAVSCFGa
**ASTC-PRP1**	KSLQKDVFVPQYDAEDHKLDVMPDDGTAETALLNYLYARQML
**ASTC-PRP2**	LSSNLDVTELQR
**SIFamide**	GYRKPTFNGSIFa
**Bursicon α**	DECKLTPVVHVLQYPGCVPKPIPSYACVGHCTSYVQVSGSKLWQTERSCMCCQESGQREASVSIFCPKAKQS DQKFRKIVTRAPVECMCRPCTQAEESKAIPQEVAGFVSGGMSLESMPFV
**Bursicon β**	ARPQCETIPSTIHITKDEFSSSGQLERTCEGDIPVNKCEGTCSSQVQPSVISPSGFNKECSCCKETGLRVREITLT RCFNPDGQQVAGDQGRLTVKLREPSDCRCSRCEN
**Calci-A**	GFDFGLGRGFSASQAAKHKMGLEAAEFPSGPa
**Calci-B**	GLDFGLGRGFSGSQAAKHMMGLAAANFAGGPa
**EH**	SIGSCVRNCGQCKSMYGRFFQGTACADACLAGNEGPDCYNPSMVSRFL
**PDH**	NSELINSLLGLPKIMNEAa
**Sulf-1**	ALDYGHLRFa
**Sulf-2**	NPDY$_{(SOH)}$GMMKFa
**Sulf-3**	SPDY$_{(SOH)}$GFMKFa
**Sulf-4**	SPDYGFMKFa
**Sulf-PRP1**	ADQEHFISSARVGPLFEDFEPDLYDYED
**Sulf-PRP2**	TASLASAGESGGQLADA
**Sulf-PRP3**	RAAKSAGRS
**TRP-1**	PHTGFLGMRa
**TRP-2**	APSGFAGMRa
**TRP-3**	VPSGFGGMRa
**TRP-4**	APQNSFLGLRa
**TRP-PRP1**	SAPAPTELQLPSVTLRQPLPGQLPSPQQLLQLFRAAGPYSRQLAARIAD
**TRP-PRP2**	EELGARGSEDHLE
**TRP-PRP3**	ADVVEGGEH
**TRP-PRP4**	EEQGHP
**Orco-1**	NFDEIDRSGFSGFA
**Orco-2**	NFDEIDRSGFTGFA
**Orco-3**	NFDEIDRSGFSGFV
**Orco-PRP1**	RPYKDQQLSDRGIDSLGGGHLLRGIDSLGGGHLLRGIDSLGGGHLLRGIDSLGGGHLLRGIDSLGGGHLLRE ADSQGG
**Orco-PRP2**	HLSNVYTQRLIDYYNSHPEAYD
**Orco-PRP3**	ALDPLSGVSFGVE
**Orco-PRP4**	LDSLSGMTFGLE
**Orco-PRP5**	PQQSSVQ
**Orco-PRP6**	EAPAKEKL
**IRP**	PVDKRGPAMHFCGPSLADALEMVCEFGMGKRSRTAPRQNLLRPEFSGGHLSTSSASTMPYYQTQLYQSADS RTKRSPGIVEECCLNPCTLSTLAQYC
**NPF**	SEPRDLRSAGSGEMLSGDIDNYLSELKDFYTKVGRPRFa

Abbreviations: "PRP" is the briefing of precursor-related peptide; "a" represents the C-terminal glycine that will be amidated during post-translational modification. (SOH) marks the amino acid residues that will be sulfidated.
