# Supplementary material for: Transcriptomic Analysis of Neuropeptides and Peptide Hormones in the Barnacle Balanus amphitrite: Evidence of Roles in Larval Settlement
Source: PLoS One. 2012 Oct 2;7(10):e46513. doi: 10.1371/journal.pone.0046513 (PMC3462748; doi:10.1371/journal.pone.0046513)
Supplement: Table S2 — Precursor sequences of neuropeptides and peptide hormones from Balanus amphitrite. Signal peptides are marked as green letter, precursor related peptides are marked as blue letter, and mature neuropeptides are marked as red letter. The putative mono-, di- or tribasic cleavage sites are underlined, and amino acid residues that predicted to be sulfated are shaded as pink. (PDF) [file pone.0046513.s002.pdf]

Table S2. Precursor sequences of neuropeptides and peptide hormones from *Balanus amphitrite*. Signal peptides are marked as green letter, precursor related peptides are marked as blue letter, and mature neuropeptides are marked as red letter. The putative mono-, di- or tribasic cleavage sites are underlined, and amino acid residues that predicted to be sulfated are shaded as pink.

A-type allatostatin

1	ATG	ATG	TCA	TGT	CGG	CTG	ATG	CTG	GCG	ACG	CTG	CTG	GCT	CTG	GCG	45
1	M	M	S	C	R	L	M	L	A	T	L	L	A	L	A	15
46	ACG	CTC	GTG	ACT	CCC	CAC	TCT	GTC	AGC	GCA	AAA	CAG	CTG	GGA	CAG	90
16	T	L	V	T	P	H	S	V	S	A	K	Q	L	G	Q	30
91	GAC	CAT	CAG	CTG	GCC	ACC	GAT	CTG	CTC	GAC	GAC	AAC	TAC	GAT	GAC	135
31	D	H	Q	L	A	T	D	L	L	D	D	N	Y	D	D	45
136	ACC	TAC	GAC	CTG	GAG	CCC	GAC	GGG	GAG	GAT	GTT	GAA	AAG	CGC	GAC	180
46	T	Y	D	L	E	P	D	G	E	D	V	E	K	R	D	60
181	CGA	ACG	TAC	GGA	TTC	GGT	CTG	GGG	AAA	CGG	GAC	CAG	AAC	CGT	CCG	225
61	R	T	Y	G	F	G	L	G	K	R	D	Q	N	R	P	75
226	TAC	CGC	GCC	TAC	GGG	TTC	GGC	CTT	GGC	AAG	CGG	GGC	CGT	CCG	TAT	270
76	Y	R	A	Y	G	F	G	L	G	K	R	G	R	P	Y	90
271	AGT	ACG	TAC	GGG	TTT	GGG	CTA	GGG	AAG	CGC	GAG	AGA	ACC	TAC	GGA	315
91	S	T	Y	G	F	G	L	G	K	R	E	R	T	Y	G	105
316	TTC	GGT	CTG	GGA	AAA	CGA	GAG	CGA	ACG	TAC	GGA	TTC	GGT	CTC	GGC	360
106	F	G	L	G	K	R	E	R	T	Y	G	F	G	L	G	120
361	AAG	CGG	CAG	CCG	TCG	TAC	GCG	TTT	GGA	CTT	GGA	AAG	CGT	CCC	TCG	405
121	K	R	Q	P	S	Y	A	F	G	L	G	K	R	P	S	135
406	GCG	TCC	CAA	TAC	GCG	TTC	GGA	CTC	GGC	AAA	CGA	AAT	GGC	CAT	GAC	450
136	A	S	Q	Y	A	F	G	L	G	K	R	N	G	H	D	150
451	GAC	ATT	CCC	AAC	AAG	CGA	CCA	ACG	TAC	GGG	TTC	GGT	CTA	GGG	AAG	495
151	D	I	P	N	K	R	P	T	Y	G	F	G	L	G	K	165
496	CGC	AGC	TGG	AGC	TGG	TTC	GAG	GGC	CCA	ACC	CGG	GAC	GTC	CTG	AAG	540
166	R	S	W	S	W	F	E	G	P	T	R	D	V	L	K	180
541	CGG	CCC	AGC	TAC	GGC	TTC	GGT	CTG	GGA	AAG	AGG	AGC	GCG	CCG	GCG	585
181	R	P	S	Y	G	F	G	L	G	K	R	S	A	P	A	195
586	GGG	GTC	GAC	GAG	GAC	CTG	CTG	GAC	GAT	CCG	GAC	ACG	GCG	CTG	CTG	630
196	G	V	D	E	D	L	L	D	D	P	D	T	A	L	L	210
631	CTG	ATG	ACG	GCA	GCG	CGG	GAC	CTG	GCT	GAT	GGG	GAG	GTC	AAG	TCG	675
211	L	M	T	A	A	R	D	L	A	D	G	E	V	K	S	225
676	CCC	GCG	GGC	CGG	CAC	AGG	ATC	GGC	TCC	GAC	TCG	GGT	CAG	ACA	CAC	720
226	P	A	G	R	H	R	I	G	S	D	S	G	Q	T	H	240
721	ACC	AAG	CCG	GAG	GAC	GTG	TCC	TCA	CAG	GAA	AAG	GGC	GTT	CGG	TTT	765
241	T	K	P	E	D	V	S	S	Q	E	K	G	V	R	F	255
766	CAG	CTC	TAG	774												
256	Q	L	*													

B-type allatostatin

1 TTG GTG CTG CTG TCG TGG AGC TGC CTG CTA CTG CTG ACG GTG CGC 45
1 L V L L S W S C L L L L T V R 15
46 GCT GGA TCG GCA GAG GCT GAA CAG CCG CAG GCT GTC TCG GCA GAC 90
16 A G S A E A E Q P Q A V S A D 30
91 AAA CGC GAC TGG AAC GCG CTT CAC GGC AAC TGG GGA AAA CGA GAC 135
31 K R D W N A L H G N W G K R D 45
136 CCG TCA GAG GTC ACC GGA GAC CCC GAG GAT GAC CGG GAG TTG ATG 180
46 P S E V T G D P E D D R E L M 60
181 CGA GAG GTC GCC AAT GGG CTG GTG TTA TCT GCA TAC GAG CCG GAG 225
61 R E V A N G L V L S A Y E P E 75
226 GAT AAG AGG AAG TGG AAC AGC TTC ACC GGC TCC TGG GGC AAG CGG 270
76 D K R K W N S F T G S W G K R 90
271 GGG GCC AAG TGG AAC GGC TTC GCT GGC TCC TGG GGA AAG CGG TCC 315
91 G A K W N G F A G S W G K R S 105
316 AAG TGG AAC GAC ATG GGC CCG TCC TGG GGC AAG AGA GCC AAG TGG 360
106 K W N D M G P S W G K R A K W 120
361 GAA AAC TTT GGC GGA TCG TGG GGA AAG CGA AGC AAG TGG GAC GAC 405
121 E N F G G S W G K R S K W D D 135
406 ATT GAC GAC GAC TCC TTG GAA AAG CGG GCA AAG TGG GAC AAC TTT 450
136 I D D D S L E K R A K W D N F 150
451 GGC GGA TCC TGG GGC AAG AGG AAG AAC TGG GAT AAC TTC AAC GGC 495
151 G G S W G K R K N W D N F N G 165
496 GCC TGG GGC AAG AGG AAA GGC TGG GAC AGC TTC AGC GGC GGA TGG 540
166 A W G K R K G W D S F S G G W 180
541 GGC AAG CGG TCA GCG TGG AAC AAC TTC GGC GGC TCG TGG GGC AAG 585
181 G K R S A W N N F G G S W G K 195
586 AGG TCC GTG TCG ACG CCA GAG CTT CAG GAG GCT GCG GAT CGT TTA 630
196 R S V S T P E L Q E A A D R L 210
631 GAA AAG TTT ATT GTA AGC CAT TAC GAA CCA TCG CCA GAA GTC GAG 675
211 E K F I V S H Y E P S P E V E 225
676 GTA TCT TCT GGT GAA ACA CAA GCG AAG AAA CGA AAA GAT TGG ACA 720
226 V S S G E T Q A K K R K D W T 240
721 CAA CTC AAC GGC ATG TGG GGA AAA CGG TCT TCA TCA AAG GCG CTT 765
241 Q L N G M W G K R S S S K A L 255
766 CTG CTG GCG TGA 777
256 L L A *

C-type allatostatin

1 ATG ACG CTA GCC CGT TTG TCG CTG CTC CTC ACC ATC TGC GTG GCC 45
1 M T L A R L S L L L T I C V A 15
46 GTG CTG GTG GCC GCC GCA GCC GGC AAG AGC CTC CAG AAG GAC GTG 90
16 V L V A A A A G K S L Q K D V 30
91 TTC GTC CCG CAG TAC GAC GCC GAG GAC CAC AAA CTG GAT GTG ATG 135
31 F V P Q Y D A E D H K L D V M 45
136 CCT GAC GAC GGC ACG GCG GAG ACG GCG CTG CTT AAC TAC CTG TAC 180
46 P D D G T A E T A L L N Y L Y 60
181 GCC CGT CAA ATG CTG CGG CGA CTC AGC AGC AAC CTG GAC GTG ACC 225
61 A R Q M L R R L S S N L D V T 75
226 GAG CTG CAG CGC AAG CGC TCC TAC TGG AAA CAG TGC TCC TTC AAC 270
76 E L Q R K R S Y W K Q C S F N 90
271 GCC GTC TCC TGC TTC GGC TAG 291
91 A V S C F G *

Bursicon-alpha

```
1      ATG AGC TGG TGC CTG GTT CTG GTG GCC GCG CTG ACG GTG GCC GCC   45
1       M   S   W   C   L   V   L   V   A   A   L   T   V   A   A    15
46     TCG CGG CTG GCG CTC ACC GGC GCC GAT GAG TGC AAA CTG ACG CCG   90
16      S   R   L   A   L   T   G   A   D   E   C   K   L   T   P    30
91     GTC GTG CAC GTG CTC CAG TAC CCG GGC TGT GTG CCC AAG CCG ATT   135
31      V   V   H   V   L   Q   Y   P   G   C   V   P   K   P   I    45
136    CCG TCG TAC GCC TGC GTC GGC CAC TGC ACC AGC TAT GTG CAG GTC   180
46      P   S   Y   A   C   V   G   H   C   T   S   Y   V   Q   V    60
181    TCC GGC AGC AAA CTG TGG CAA ACG GAG CGC TCC TGC ATG TGC TGC   225
61      S   G   S   K   L   W   Q   T   E   R   S   C   M   C   C    75
226    CAA GAG AGT GGC CAG CGA GAG GCC TCC GTC TCA ATC TTC TGC CCC   270
76      Q   E   S   G   Q   R   E   A   S   V   S   I   F   C   P    90
271    AAG GCC AAA CAA AGC GAC CAA AAG TTC CGA AAG ATC GTG ACC CGG   315
91      K   A   K   Q   S   D   Q   K   F   R   K   I   V   T   R    105
316    GCG CCG GTC GAG TGC ATG TGC CGG CCG TGC ACC CAG GCC GAG GAG   360
106     A   P   V   E   C   M   C   R   P   C   T   Q   A   E   E    120
361    AGC AAG GCC ATC CCG CAG GAG GTG GCC GGC TTC GTC TCC GGC GGT   405
121     S   K   A   I   P   Q   E   V   A   G   F   V   S   G   G    135
406    ATG TCG CTC GAG TCG ATG CCG TTC GTC TGA   435
136     M   S   L   E   S   M   P   F   V   *
```

Bursicon-beta

```
1      ATG GCC CGC CTC CGC GCC GCC GGC CTG CTG CTA GCG CTG CTG ACC   45
1       M   A   R   L   R   A   A   G   L   L   L   A   L   L   T    15
46     GTC AGC GGC TGC CGC GCT GCC CGG CCG CAG TGT GAG ACC ATC CCC   90
16      V   S   G   C   R   A   A   R   P   Q   C   E   T   I   P    30
91     TCG ACC ATA CAC ATC ACC AAA GAT GAG TTC AGC TCC TCT GGC CAG   135
31      S   T   I   H   I   T   K   D   E   F   S   S   S   G   Q    45
136    CTG GAG CGG ACG TGC GAG GGC GAC ATT CCT GTC AAC AAG TGC GAA   180
46      L   E   R   T   C   E   G   D   I   P   V   N   K   C   E    60
181    GGC ACG TGC AGC TCC CAG GTG CAG CCG TCG GTC ATC TCG CCG TCC   225
61      G   T   C   S   S   Q   V   Q   P   S   V   I   S   P   S    75
226    GGC TTC AAT AAG GAG TGC AGC TGC TGT AAG GAG ACC GGT CTG CGC   270
76      G   F   N   K   E   C   S   C   C   K   E   T   G   L   R    90
271    GTG CGC GAG ATC ACG CTG ACG CGC TGC TTC AAC CCG GAC GGT CAG   315
91      V   R   E   I   T   L   T   R   C   F   N   P   D   G   Q    105
316    CAG GTG GCC GGC GAC CAG GGC CGG CTG ACG GTC AAA CTG CGC GAG   360
106     Q   V   A   G   D   Q   G   R   L   T   V   K   L   R   E    120
361    CCC TCC GAC TGC CGC TGT TCG CGC TGT GAA AAC TAG   396
121     P   S   D   C   R   C   S   R   C   E   N   *
```

Calcitonin-like diuretic hormone A

```
1      ATG GTG CGG CAC GTG ACT GGG CTG GGA GCA GTG GCC GCG CTG GCC   45
1       M   V   R   H   V   T   G   L   G   A   V   A   A   L   A    15
46     GCG CTC GTC TGC TTC GCA CTC TGC TGG GTC TCA GCC TCT GCC GCC   90
16      A   L   V   C   F   A   L   C   W   V   S   A   S   A   A    30
91     AGT CTC CAC CTG GAC GAT GGA GAA AGA AAA CCA TGG ATG GAC GAA   135
31      S   L   H   L   D   D   G   E   R   K   P   W   M   D   E    45
136    ATG GAG GCC AAC CTA CAG AAC GAC CTC TAC CTG AGC GAG CTG GTC   180
46      M   E   A   N   L   Q   N   D   L   Y   L   S   E   L   V    60
181    AGC CGG CTG CGC GCG CTC AAC GAC GCC GAG GTG GCC GGA CTC GCC   225
61      S   R   L   R   A   L   N   D   A   E   V   A   G   L   A    75
226    CAC AAG CGC GGT TTT GAC TTC GGC CTG GGC CGC GGA TTT TCG GCC   270
76      H   K   R   G   F   D   F   G   L   G   R   G   F   S   A    90
271    TCC CAG GCC GCC AAG CAC AAG ATG GGT CTG GAG GCG GCT GAG TTC   315
91      S   Q   A   A   K   H   K   M   G   L   E   A   A   E   F    105
316    CCC AGC GGC CCC GGA CGC AGG CGG CGC TCC CTG GAG CAA GAC CTG   360
106     P   S   G   P   G   R   R   R   R   S   L   E   Q   D   L    120
361    AGG CAC TAG   369
121     R   H   *
```

Calcitonin-like diuretic hormone B

```
1      ATG TGC CGC TCT GCG CTG CTG TCC GGC GGT GTG ACC GTG TTC CTC   45
1       M   C   R   S   A   L   L   S   G   G   V   T   V   F   L    15
46     GTC GCC CTG GTC CTC TGC ACA CTG TCA CTC TGC TCA GCG GCC AGC   90
16      V   A   L   V   L   C   T   L   S   L   C   S   A   A   S    30
91     CTA CAG GTC AAC GAT GGA CTT GAA AGG AAG CCG TGG ATG GAT GCG   135
31      L   Q   V   N   D   G   L   E   R   K   P   W   M   D   A    45
136    GTA CAG CGA GAA CTG CCC AGT GAC GCC TAC ATG ACC GAG TTT ATC   180
46      V   Q   R   E   L   P   S   D   A   Y   M   T   E   F   I    60
181    GCC CGG CTG CGA GCG CTC AAA GAC GCC GAG GAC ATC GGG CTT GCA   225
61      A   R   L   R   A   L   K   D   A   E   D   I   G   L   A    75
226    CAG AAG CGC GGA CTG GAC TTC GGC CTG GGA CGC GGC TTC TCC GGC   270
76      Q   K   R   G   L   D   F   G   L   G   R   G   F   S   G    90
271    AGC CAG GCG GCG AAG CAC ATG ATG GGA CTG GCG GCG GCC AAC TTC   315
91      S   Q   A   A   K   H   M   M   G   L   A   A   A   N   F    105
316    GCC GGC GGT CCG GGC CGG CGG CGG CGG TCA GCC CCT CTG CCG ACG   360
106     A   G   G   P   G   R   R   R   R   S   A   P   L   P   T    120
361    CTC CAC TAG   369
121     L   H   *
```

Eclosion hormone

```
1     ATG AAC AGG ACC GAG TCG CTG CTG GTG CTC TGC GGC GCG ATT GTC   45
1      M   N   R   T   E   S   L   L   V   L   C   G   A   I   V    15
46    GTC CTC CTG CTG AGC GGC CCG GCC CAG GCC AGC ATC GGC TCT TGT   90
16     V   L   L   L   S   G   P   A   Q   A   S   I   G   S   C    30
91    GTC CGA AAC TGC GGC CAG TGC AAG AGC ATG TAC GGC CGG TTC TTC   135
31     V   R   N   C   G   Q   C   K   S   M   Y   G   R   F   F    45
136   CAG GGC ACC GCC TGT GCG GAC GCC TGT CTG GCC GGG AAT GAG GGC   180
46     Q   G   T   A   C   A   D   A   C   L   A   G   N   E   G    60
181   CCC GAC TGC TAC AAC CCC TCG ATG GTC TCG CGC TTT CTG AAG CGG   225
61     P   D   C   Y   N   P   S   M   V   S   R   F   L   K   R    75
226   CGA GCC TCG CCG GTG GTC CCC GCG TCC CCC GGG CTG GCG TCC CTC   270
76     R   A   S   P   V   V   P   A   S   P   G   L   A   S   L    90
271   GGC GGC AGT ACG AGT ACT ACA GCG TGG ACG GCC AGC CGG CAG AGG   315
91     G   G   S   T   S   T   T   A   W   T   A   S   R   Q   R    105
316   TGC CCA TTT CCG GCA GCT CGG CTC TCT TCA GAA TGC TCT CAG CTC   360
106    C   P   F   P   A   A   R   L   S   S   E   C   S   Q   L    120
361   GAG GAG TGG AGG CGC GGA AGT ACG CCC CGG CGA GCT CTG CAC CTC   405
121    E   E   W   R   R   G   S   T   P   R   R   A   L   H   L    135
406   CAT TAC GCA TTT CCC ATC GTG AAC TGA   432
136    H   Y   A   F   P   I   V   N   *
```

Insulin-related peptide

```
1     ATG GCG CAG CTG CGA CAC GTC CTC TTC ATG ATG CTG CTC GGA GCC   45
1      M   A   Q   L   R   H   V   L   F   M   M   L   L   G   A    15
46    GTT TCC ACG GAC ATT GCA TCG GGT TCC AGC AGC TGG AAG CGG CCC   90
16     V   S   T   D   I   A   S   G   S   S   S   W   K   R   P    30
91    GTC GAC AAG CGT GGT CCT GCG ATG CAC TTC TGC GGT CCC TCG CTG   135
31     V   D   K   R   G   P   A   M   H   F   C   G   P   S   L    45
136   GCT GAC GCC CTG GAA ATG GTG TGC GAG TTC GGA ATG GGA AAA CGG   180
46     A   D   A   L   E   M   V   C   E   F   G   M   G   K   R    60
181   TCC AGA ACC GCG CCT CGC CAG AAT CTG CTG AGG CCG GAA TTT TCT   225
61     S   R   T   A   P   R   Q   N   L   L   R   P   E   F   S    75
226   GGC GGA CAT CTC TCC ACC TCG TCT GCT TCC ACC ATG CCC TAC TAC   270
76     G   G   H   L   S   T   S   S   A   S   T   M   P   Y   Y    90
271   CAG ACG CAG CTC TAC CAG TCT GCA GAC TCC AGG ACC AAG AGG AGT   315
91     Q   T   Q   L   Y   Q   S   A   D   S   R   T   K   R   S    105
316   CCG GGC ATT GTG GAG GAA TGC TGC CTG AAT CCG TGC ACG CTG AGC   360
106    P   G   I   V   E   E   C   C   L   N   P   C   T   L   S    120
361   ACA CTA GCA CAG TAC TGC TGA   381
121    T   L   A   Q   Y   C   *
```

Neuropeptide F

```
1     ATG AGG GAC AGC ATG CGA CTG GCG CTG GCA CTA CTG CTC ACG GCT   45
1      M   R   D   S   M   R   L   A   L   A   L   L   L   T   A    15
46    CTG CTG GTG CTG CCC AGA CAT CAC GTG ACC GCC TCG GAG CCA CGT   90
16     L   L   V   L   P   R   H   H   V   T   A   S   E   P   R    30
91    GAC CTG AGG TCG GCC GGC AGC GGC GAG ATG CTA TCC GGC GAC ATC   135
31     D   L   R   S   A   G   S   G   E   M   L   S   G   D   I    45
136   GAC AAC TAC CTC AGC GAG CTC AAG GAT TTC TAC ACG AAA GTC GGC   180
46     D   N   Y   L   S   E   L   K   D   F   Y   T   K   V   G    60
181   AGG CCC AGG TTC GGC AAA CGC AGC ACC CGC GTC CGG CGG TGG CGC   225
61     R   P   R   F   G   K   R   S   T   R   V   R   R   W   R    75
226   ACG GTG GCG TCA CTC GGT GAC GTC ATA CAC GAG CTG CGC TTC TGA   270
76     T   V   A   S   L   G   D   V   I   H   E   L   R   F   *
```

Orcokinin

```
1     ATG GCG AGT CTG TCC TAC TCG GCT CTG CTG CTG GCC ACA GCC CTG   45
1      M   A   S   L   S   Y   S   A   L   L   L   A   T   A   L   15
46    GTG GGC CTG GAG GCG CGC CCC TAC AAG GAC CAG CAG CTA TCG GAC   90
16     V   G   L   E   A   R   P   Y   K   D   Q   Q   L   S   D   30
91    CGG GGG ATC GAC TCT CTG GGC GGC GGC CAC TTA CTC AGA GGT ATC   135
31     R   G   I   D   S   L   G   G   G   H   L   L   R   G   I   45
136   GAC TCA CTG GGC GGC GGC CAC CTG TTG AGG GGA ATC GAC TCT CTC   180
46     D   S   L   G   G   G   H   L   L   R   G   I   D   S   L   60
181   GGC GGA GGA CAC CTC CTG AGA GGA ATC GAC TCC CTG GGC GGA GGA   225
61     G   G   G   H   L   L   R   G   I   D   S   L   G   G   G   75
226   CAC CTG TTG CGA GGA ATC GAC TCT CTG GGA GGA GGA CAT CTG CTC   270
76     H   L   L   R   G   I   D   S   L   G   G   G   H   L   L   90
271   CGG GAG GCG GAC TCC CAG GGA GGA CGG CGG CAC CTC TCC AAC GTC   315
91     R   E   A   D   S   Q   G   G   R   R   H   L   S   N   V   105
316   TAC ACA CAG AGG CTG ATC GAC TAC TAC AAC AGC CAC CCG GAG GCC   360
106    Y   T   Q   R   L   I   D   Y   Y   N   S   H   P   E   A   120
361   TAT GAC CGG GAG GTG CGC GCC CTG GAC CCG CTC AGC GGC GTC TCG   405
121    Y   D   R   E   V   R   A   L   D   P   L   S   G   V   S   135
406   TTC GGT GTG GAG AAG CGG CTC GAC TCG CTC AGT GGC ATG ACC TTC   450
136    F   G   V   E   K   R   L   D   S   L   S   G   M   T   F   150
451   GGC CTG GAG AAG CGC AAC TTT GAC GAA ATC GAT CGA TCG GGC TTC   495
151    G   L   E   K   R   N   F   D   E   I   D   R   S   G   F   165
496   AGC GGC TTC GCG AAG CGC AAC TTC GAC GAG ATC GAC CGA TCT GGC   540
166    S   G   F   A   K   R   N   F   D   E   I   D   R   S   G   180
541   TTC ACT GGC TTT GCG AAG CGG CCA CAG CAG TCC AGC GTA CAA AAG   585
181    F   T   G   F   A   K   R   P   Q   Q   S   S   V   Q   K   195
586   CGC AAC TTT GAC GAA ATC GAT CGC AGC GGA TTC AGC GGT TTT GTG   630
196    R   N   F   D   E   I   D   R   S   G   F   S   G   F   V   210
631   AAG CGG GAG GCG CCG GCC AAA GAG AAA CTG TAG   663
211    K   R   E   A   P   A   K   E   K   L   *
```

Pigment dispersing hormone

```
1     ATG AGG ACC GGT ATC GTC TGT CTG CTG CTG AGC GCG CTG GTC GCC   45
1      M   R   T   G   I   V   C   L   L   L   S   A   L   V   A   15
46    CTC GCC CTC GGC GCG CCC GAC CGG TAC TCC AGC GAC GAG CTC GAC   90
16     L   A   L   G   A   P   D   R   Y   S   S   D   E   L   D   30
91    CTG GCC ACG CGA CAG ATG ATC GGT GAG CTG GCC GGC CGA ATC CTG   135
31     L   A   T   R   Q   M   I   G   E   L   A   G   R   I   L   45
136   CGC CTG GCC CGC CCC GCC AGC GCC CTG GGC GCG CAG AAA CGC AAC   180
46     R   L   A   R   P   A   S   A   L   G   A   Q   K   R   N   60
181   TCG GAG CTC ATC AAC TCG CTG CTC GGC CTG CCC AAG ATC ATG AAC   225
61     S   E   L   I   N   S   L   L   G   L   P   K   I   M   N   75
226   GAG GCC GGC CGG TGA   240
76     E   A   G   R   *
```

SIFamide

```
1     ATG GGC AGC CGG TGC GCA GTT CGT CGA GTG GCC GCC GTG CTG GTG   45
1      M   G   S   R   C   A   V   R   R   V   A   A   V   L   V   15
46    GTG GCG CTA GTA GCG ATG GCG CTA CTA GCG CCT CTG ACA GAG GCC   90
16     V   A   L   V   A   M   A   L   L   A   P   L   T   E   A   30
91    GGC TAC CGC AAG CCA ACA TTC AAC GGC AGC ATT TTC GGC AAA CGC   135
31     G   Y   R   K   P   T   F   N   G   S   I   F   G   K   R   45
```

```
136    GCA GCC GCC GCG GCC GAA GCG GAA GCA GCC CAG GGC CTG GCC CGC   180
46      A   A   A   A   A   E   A   E   A   A   Q   G   L   A   R    60
181    ATG TGT GCA GCC GCC TAT ACA GTC TGT GGT TTT CCC GCC GAA TAA   225
61      M   C   A   A   A   Y   T   V   C   G   F   P   A   E   *
```

Sulfakinin

```
1      ATG TTG GTC GAG GGC TGT CTA CCT GTG ATC CTG ATG ATA GCC GTG   45
1       M   L   V   E   G   C   L   P   V   I   L   M   I   A   V    15
46     CTG TCC GGG CGG CAA CAC CTG GTC AGG GCC GAC CAG GAG CAC TTC   90
16      L   S   G   R   Q   H   L   V   R   A   D   Q   E   H   F    30
91     ATC AGT AGC GCG CGC GTC GGA CCC CTG TTC GAG GAC TTT GAG CCA   135
31      I   S   S   A   R   V   G   P   L   F   E   D   F   E   P    45
136    GAC CTG TAC GAC TAC GAG GAC AAG CGG GCG CTC GAC TAC GGC CAC   180
46      D   L   Y   D   Y   E   D   K   R   A   L   D   Y   G   H    60
181    CTG CGC TTC GGC AAG CGG ACG GCG AGC CTC GCG TCC GCC GGA GAG   225
61      L   R   F   G   K   R   T   A   S   L   A   S   A   G   E    75
226    TCC GGC GGA CAG CTC GCC GAC GCC AAA CGC AAC CCC GAC TAC GGC   270
76      S   G   G   Q   L   A   D   A   K   R   N   P   D   Y   G    90
271    ATG ATG AAG TTT GGC CGG CGC TCC CCG GAT TAC GGC TTC ATG AAG   315
91      M   M   K   F   G   R   R   S   P   D   Y   G   F   M   K    105
316    TTC GGT CGA CGC TCG CCA GAC TAC GGC TTC ATG AAG TTC GGA AAG   360
106     F   G   R   R   S   P   D   Y   G   F   M   K   F   G   K    120
361    AGG CGA GCG GCA AAA TCG GCC GGT AGG AGC TGA   393
121     R   R   A   A   K   S   A   G   R   S   *
```

Tachykinin-related peptide

```
1      ATG CAG AGC GTG TCG CCG CTG CCG CTG CTG GCC CTC AGT CTG CTG   45
1       M   Q   S   V   S   P   L   P   L   L   A   L   S   L   L    15
46     GTG CTG TCT GCC GGT GCC AGC GCC GCC TCG CTG CGC CGG CCC CAC   90
16      V   L   S   A   G   A   S   A   A   S   L   R   R   P   H    30
91     ACC GGC TTC CTG GGG ATG CGC GGC AAG CGC TCG GCG CCG GCG CCG   135
31      T   G   F   L   G   M   R   G   K   R   S   A   P   A   P    45
136    ACC GAG CTG CAG CTG CCG TCC GTG ACG CTG CGC CAG CCG CTG CCC   180
46      T   E   L   Q   L   P   S   V   T   L   R   Q   P   L   P    60
181    GGC CAG CTG CCG TCG CCC CAG CAG CTG TTG CAG CTG TTC CGC GCA   225
61      G   Q   L   P   S   P   Q   Q   L   L   Q   L   F   R   A    75
226    GCC GGC CCC TAC AGC CGA CAG CTG GCC GCA CGG ATC GCC GAC AAA   270
76      A   G   P   Y   S   R   Q   L   A   A   R   I   A   D   K    90
271    CGG GCA CCC AGC GGG TTC GCC GGA ATG CGG GGA CGC AAG AGG GAG   315
91      R   A   P   S   G   F   A   G   M   R   G   R   K   R   E    105
316    GAG CTG GGC GCG CGG GGC AGT GAG GAC CAC CTG GAG AAA CGC GTG   360
106     E   L   G   A   R   G   S   E   D   H   L   E   K   R   V    120
361    CCC AGT GGC TTC GGA GGC ATG AGG GGC AAG AAG GCC GAC GTG GTG   405
121     P   S   G   F   G   G   M   R   G   K   K   A   D   V   V    135
406    GAG GGA GGG GAG CAC AAG AGG GCG CCG CAA AAC TCC TTC CTC GGA   450
136     E   G   G   E   H   K   R   A   P   Q   N   S   F   L   G    150
451    CTG AGG GGG AGG AGA GAG GAA CAG GGA CAC CCC TAA   486
151     L   R   G   R   R   E   E   Q   G   H   P   *
```
