# Supplementary material for: Transcriptomic Analysis of Neuropeptides and Peptide Hormones in the Barnacle Balanus amphitrite: Evidence of Roles in Larval Settlement
Source: PLoS One. 2012 Oct 2;7(10):e46513. doi: 10.1371/journal.pone.0046513 (PMC3462748; doi:10.1371/journal.pone.0046513)
Supplement: Table S3 — Primers used for real-time PCR amplification. (PDF) [file pone.0046513.s003.pdf]

Table S3. Primers used for real-time PCR amplification.

peptide	Forward Primer	Reverse Primer
A-type allatostatin	CAGACGGGGAGGATGTTG	AAACCCGTACGTACTATA
B-type allatostatin	TGATTGGTGCTGACAGAACAAA	ATCGCCTCTGACCTGCTGGTAT
C-type allatostatin	AAGAGCCTCCAGAAGGACGTGT	GATGTTTGCCGTTAGGGATG
Bursiconβ	ATTCCTGTCAACAAGTGCGAA	GCTAGTTTTCACAGCGCGAA
Calcitonin-A	AAAACCATGGATGGACGAAA	CTCCAGACCCATCTTGTGCT
Calcitonin-B	GCCTCTCCAGAATCATGTGC	GATGTCCTCGGCGTCTTTGA
Eclosion hormone	CGATGGTCTCGCGCTTTCTGAA	ATGGGAAATGCGTAATGGAG
Insulin-related peptide	CTCTCCACCTCGTCTGCTTC	TCCATCAGCAGTACTGTGCTAG
Neuropeptide F	CACTACTGCTCACGGCTCTG	CTGCCGACTTTCGTGTAGAA
Orcokinin	TACAAGGACCAGCAGCTATCGGA	TCCTCCTCCCAGAGAGTCGATT
Pigment dispersing hormone	AGCCACCATGAGGACCGGTAT	CAGCTCACCGATCATCTGT
SIFamide	TTGTTGACCCAAGCATATCG	CTCAGTCTCGGGGTCAGAAG
Sulfakinin	GACATTGGATGCAAGACAGAA	TGAGGTGGCGCATCGTCCTTCA
Tachykinin-related peptide	GCCTCTCCAGAATCATGTGC	GATGTCCTCGGCGTCTTTGA
